# Muscle signals to the rescue

**DOI:** 10.7554/eLife.64587

**Published:** 2020-12-21

**Authors:** Arely V Diaz, Tânia Reis

**Affiliations:** 1 Department of Medicine, University of Colorado Anschutz Medical CampusAuroraUnited States

**Keywords:** obesity, myokine, PDGF, mTOR, hepatocyte, VEGF, *D. melanogaster*

## Abstract

The skeletal muscle of fruit flies communicates with other organs to prevent the accumulation of too much fat and to protect adults against obesity.

**Related research article** Ghosh AC, Tattikota SG, Liu Y, Comjean A, Hu Y, Barrera V, Ho Sui SJ, Perrimon N. 2020. *Drosophila* PDGF/VEGF signaling from muscles to hepatocyte-like cells protects against obesity. *eLife* **9**:e56969. doi: 10.7554/eLife.56969

When indulging in a tasty meal, we rarely consider the amount of communication that takes place between various organs in our bodies. A multitude of signals flow between the digestive system and the brain, which regulate our appetite and help the body to metabolize foods by extracting nutrients and converting them into energy. However, much remains unknown about the molecular signaling pathways that regulate this internal conversation.

Fruit flies use many of the same mechanisms as humans to regulate food intake and balance energy levels, and are therefore a popular model for studying metabolism and metabolic diseases (Doane, 1960; Baker and Thummel, 2007; Musselman and Kühnlein, 2018). By manipulating the activity of individual genes in specific tissues, researchers have been able to gain more insight into how organs communicate on a molecular level. For example, it emerged that skeletal muscle – as opposed to cardiac and smooth muscle – is the source of a variety of metabolic signals that induce changes in other tissues, some of which have been found to affect the storage of fat (Benatti and Pedersen, 2014; Fisher and Maratos-Flier, 2015; Eckel, 2019). Now, in eLife, Arpan Ghosh, Norbert Perrimon and colleagues – who are based at Harvard Medical School and the Harvard Chan Bioinformatics Core – report that a signaling molecule released by skeletal muscle can protect flies from becoming obese (Ghosh et al., 2020).

The researchers studied a molecule called Pvf, which is the equivalent of two signaling factors that regulate cell growth and division in humans, called PDGF and VEGF. Fruit flies have multiple versions of the Pvf protein, which Ghosh et al. deactivated in various tissues. The experiments revealed that one of these subtypes, called Pvf1, protects adult flies against obesity by preventing excess fat from accumulating in storage sites (the fly adipose tissue) and in specialized cells called oenocytes. Oenocytes help the body absorb and break down fats to provide additional energy to cells in times of stress (Makki et al., 2014).

Ghosh et al. found that oenocytes contained high numbers of PvR, the receptor for Pvf1. When the receptor in these cells was deactivated, the flies stored more fat, similar to what was observed when the production of Pvf1 was turned off in the skeletal muscle. This suggests that communication between the muscle and oenocytes via Pvf1 and its receptor PvR is required to regulate the fat metabolism in the adult fly.

Further experiments showed that blocking a specific signaling pathway (called PI3K/Akt1/TOR) in oenocytes also led to an accumulation of fats, while reactivating the pathway prevented it. Moreover, when Pvf1 was turned off in the skeletal muscle, the signaling pathway in the oenocytes was also impaired. To better understand the role of this pathway in fat mobilization and synthesis, the researchers measured the rates of fat release from stores and new fat synthesis. Blocking this pathway had no effect on mobilizing fat from fat stores. Instead, fat synthesis increased, although the underlying mechanisms remain unclear.

To find out if this communication pathway is more important at a specific point in a fly’s life, Ghosh et al. turned their attention to young adult flies. During this age, the animals rapidly accumulate fat before achieving the steady, balanced level characteristic of mature adults. Indeed, the production of Pvf1 in the adult muscle peaked around the time when adult fat stores in the adipose tissue reached a steady-state capacity. However, when the flies were genetically modified to produce Pvf1 in the muscle earlier than normal and at higher levels, the young adults failed to store as much fat. This suggests that Pvf1 needs to be produced at a specific time and quantity to help young adult flies accumulate the right amount of fat.

Taken together, these findings show that Pvf1 released from skeletal muscle helps to end the normal increase of fat stores in young adult flies. This slows down the production of new fat, thus preventing obesity in mature adults. These results provide new insights into how internal organs communicate with each other to maintain energy levels. Further studies are needed to better understand how this muscle-derived signaling cascade regulates fat accumulation in vertebrates.
